# Influenza Vaccination in Spain: Understanding the Impact of Suboptimal Coverage on Health, Society, and the Economy

**DOI:** 10.3390/vaccines14060482

**Published:** 2026-05-28

**Authors:** María Ángeles Onieva-García, Irene Rivero-Calle, Ángel Gil de Miguel, Antoni Trilla, Alberto Pérez-Rubio

**Affiliations:** 1 Preventive Medicine and Public Health Unit, Hospital Universitario Reina Sofía, 14004 Cordoba, Spain; mariaa.onieva.sspa@juntadeandalucia.es; 2 Maimonides Biomedical Research Institute of Cordoba (IMIBIC), 14004 Cordoba, Spain; 3 Department of Medical and Surgical Sciences, University of Cordoba, 14002 Cordoba, Spain; 4 Translational Paediatrics and Infectious Diseases Section, Paediatrics Department, Hospital Clínico Universitario de Santiago de Compostela, 15706 Santiago de Compostela, Spain; 5 Genetics, Vaccines, and Infections Research Group (GENVIP), Healthcare Research Institute of Santiago de Compostela, University of Santiago de Compostela, 15706 Santiago de Compostela, Spain; 6 CIBER of Respiratory Diseases (CIBERES), Instituto de Salud Carlos III, 28029 Madrid, Spain; angel.gil@urjc.es; 7 Preventative Medicine and Public Health Area, Universidad Rey Juan Carlos, 28922 Madrid, Spain; 8 Preventive Medicine and Epidemiology Unit, Hospital Clinic, 08036 Barcelona, Spain; atrilla@clinic.cat; 9 School of Medicine and Health Sciences, University of Barcelona, 08036 Barcelona, Spain; 10 ISGlobal Research Institute, 08003 Barcelona, Spain; 11 Complejo Asistencial de Ávila, 05071 Ávila, Spain; albertoprz@gmail.com

**Keywords:** influenza vaccination, vaccine uptake, vaccination coverage, influenza burden, immunization policy, vaccine hesitancy, at-risk groups, Spain

## Abstract

Influenza is an acute viral respiratory infection that generates a substantial clinical and socioeconomic burden, especially among high-risk populations such as older adults. In Spain, influenza accounts for approximately €128 million in direct healthcare costs each season, with 80% of expenditures concentrated in individuals over 45 years of age. Vaccination remains the most effective intervention to prevent complications and reduce healthcare pressure. However, current coverage rates are consistently below international targets and continue to decline. In this context, there is an urgent need to strengthen vaccination uptake, especially among high-risk groups. Increasing vaccination uptake is essential to lessen the clinical, societal, and economic burden of influenza, since modest improvements in coverage have a significant impact on public health outcomes and economic resilience. In fact, previously published modeling analyses indicate that a 1% reduction in national vaccination coverage could result in over 6400 additional influenza cases and an additional economic burden of €1.54 million per season. Furthermore, increasing coverage to 75% in Spain could prevent approximately 180,300 additional cases—equivalent to 20% of the total—which would translate into potential savings of €43 million (€26 million in direct medical costs and €17 million in work absenteeism costs). This manuscript provides an overview of the influenza burden and vaccination landscape in Spain, focusing on clinical, societal, and economic implications of suboptimal coverage, as well as the current challenges and opportunities to improve uptake. Available evidence indicates that influenza vaccination reduces severe outcomes and healthcare burden, suggesting that mildly improving coverage in Spain could yield substantial health and economic benefits. Taken together, these findings support influenza vaccination as a public health priority and a relevant investment for health systems.

## 1. Background

Influenza is a highly transmissible acute viral respiratory infection associated with substantial morbidity, mortality, and healthcare burden, particularly among high-risk populations. Beyond its clinical impact, influenza poses a significant societal and economic burden. Strengthening seasonal vaccination coverage remains a central public health strategy to mitigate the impact of influenza.

This article provides an overview of the seasonal influenza burden and vaccination landscape in Spain, with a focus on current coverage gaps, their public health implications, and opportunities to improve uptake. It is aimed at contributing to the debate on influenza vaccination as a public health priority, by gathering published evidence and relevant official sources on influenza burden, vaccination impact, and implementation challenges in Spain.

## 2. The Impact of Influenza

### 2.1. Epidemiology

Seasonal influenza epidemiology exhibits substantial year-to-year variability, largely influenced by population-level immunity and the characteristics of circulating viral strains, which confound accurate estimations of the true disease burden. Influenza antigenic variations, together with the absence of pre-existing immunity in individuals, are often associated with higher severity and elevated mortality rates [1]. Overall, seasonal influenza affects 5–10% of the global adult population and 20–30% of children annually [2]. According to the Global Burden of Disease (GBD) study, influenza was responsible for 36.4 million (95% CI 34.2–38.7) episodes worldwide in 2019 [3].

In Spain (population: 48 m inhabitants in 2024), available data estimates an average annual incidence of 2069 influenza cases per 100,000 inhabitants [4].

In 2019 influenza ranked as the third most frequent cause of lower respiratory infections (LRI) cases worldwide, following *S. pneumoniae* and the aggregate of “all other viruses except influenza and respiratory syncytial virus (RSV)” [3]. From 2019 to 2021, the global incidence of influenza decreased substantially, as well as the incidence of other infectious respiratory diseases. It was mainly due to the reduction in respiratory viruses’ transmission due to non-pharmacological interventions implemented during the COVID-19 pandemic [3]. Nevertheless, preliminary estimates for the 2023/2024 season indicate that influenza incidence returned to pre-COVID-19 pandemic patterns [5].

According to the acute respiratory infection surveillance system (SiVIRA) report from the Instituto de Salud Carlos III, during the 2022–2023 season, lower incidence rates of acute respiratory infections (ARIs) in Primary Care (PC) and severe acute respiratory infections (SARIs) in hospitals were observed compared to the previous season (2021–2022). During the 2022–2023 season, the incidence of ARI consultations concurred with the first seasonal wave of COVID-19, influenza and RSV activity, particularly affecting children under four years of age. The incidence of respiratory was highest among vulnerable populations. The estimated incidence of influenza in PC was highest among children under 15 years of age. Similarly, the estimated incidence rate of COVID-19 in PC was highest among children under 5 years of age (very unstable estimates) and in the group aged 65 and older. Influenza and COVID-19 also contributed significantly to hospitalizations, especially among older adults aged 79 and older [6].

### 2.2. Clinical Burden

Influenza is a major contributor to global morbidity and mortality, causing 3 to 5 million cases of severe infection, and between 290,000 and 650,000 respiratory deaths annually, as estimated by the World Health Organization (WHO) [7]. In Spain, available data estimated 1540 deaths in 2023 [8].

Seasonal influenza and its complications result in a considerable clinical burden, increased hospitalizations and outpatient visits, placing considerable strain on healthcare systems, which may be overwhelmed during peak periods of viral circulation. In Spain, over the 2015–2020 period, 145,255 hospitalizations were recorded, with 7.8% of those requiring ICU admission (11,367 ICU admissions), and an average of 1600 deaths per season [9]. Certain population groups are particularly vulnerable to complications, age being among the most significant contributors to increased risk of complications. Notably, up to 60% of hospitalizations occurred in patients >65 years old and 82% of deaths were also reported in this age group. Significantly, differences between seasons were notable, mainly due to antigenic variability and differences in immunization rates [9]. Other studies focused on the same period in Spain reported a lower number of hospitalizations (127,160), although they reported similar rates of hospitalizations requiring ICU admission (7.4%). Again, the highest burden of hospitalization, longest median length of stay, and greatest mortality was observed among the elderly [10]. Children are the second most affected group, with hospitalization rates attributable to influenza of 251 per 100,000 in children <5 years old on average per season [11]. In Spain, the highest cumulative incidence rate for influenza is consistently observed in the 0–4 age group, followed by the 5–14 age group [12].

The risks of severe complications, hospital admission, and death are higher among the elderly (>65 years old) and in children under 5 years old [13]. Beyond age, additional groups are particularly vulnerable to influenza infection, either due to a higher risk of complications, or increased exposure and risk of infection.

In pregnant women, the complications of influenza are associated with a higher probability of hospitalization compared to non-pregnant women [14]. Furthermore, individuals with chronic conditions—including cardiovascular, pulmonary, renal, metabolic, neurodevelopmental, liver or hematologic diseases—as well as immunosuppressed individuals are also at greater risk of severe illness or complications [15]. In particular, among patients with chronic obstructive pulmonary disease (COPD), influenza infection has been associated with an increased risk of ischemic stroke, pneumonia, respiratory failure, and COPD exacerbations [16]. According to data from US Medicare, patients with COPD are twice as likely to be hospitalized for influenza as patients without COPD, with a median length of hospital stay of 6 days. Hospitalized patients were more likely to have comorbidities, and 34% were admitted to the intensive care unit (ICU) [17]. Moreover, in patients with diabetes, influenza is associated with more severe complications entailing higher hospitalization rates, pneumonia, and mortality, compared to individuals without diabetes [18].

In addition, influenza has long been associated with increased cardiovascular (CV) risk, with an estimated population attributable risk of 3.9% for coronary artery disease (CAD) [19].

Finally, given their critical role in patient care and their frequent contact with potentially infected individuals, healthcare professionals (HCPs) are at higher risk of infection when compared to adults working in non-healthcare settings because they are frequently exposed to the influenza virus [20]. More importantly, HCPs are also at higher risk of transmitting influenza to vulnerable populations.

### 2.3. Socioeconomic Burden

Influenza symptoms generally persist for up to 10 days before full recovery. During this period patients are advised to remain at home both to facilitate recovery and to prevent further viral transmission [21]. Consequently, influenza substantially disrupts individuals’ ability to perform daily activities, particularly work and school, thereby imposing a significant burden on employees, employers, and society at large [22,23]. Influenza/influenza-like illness (ILI) are associated with high levels of work absenteeism among adults, reducing productivity due to time away from work (mean time out of work of 2–3 days, ranging from <1 to >10 days) and reduced performance at work [22,23]. Likewise, influenza negatively impacts school attendance, leading to several days of missed classes among children and adolescents [24].

Data from the 2009 H1N1 pandemic in Spain demonstrate a significant reduction in Health-Related Quality of Life (HRQoL) among influenza patients. Using the EQ-5D index, inpatients experienced a mean HRQoL loss of 0.58 and outpatients a loss of 0.43 [25], with pain/discomfort and limitations in usual activities most affected. While most patients recovered after discharge, the societal burden was substantial, with losses estimated at 6777 QALYs for outpatients, 94 for inpatients, and an additional 11,981 QALYs from fatal cases—exceeding the burden reported for some other acute infectious diseases [25].

Influenza and ILI infections affect not only patients, but also their caregivers. A systematic review reported 0.5–10.7 days out of work among caregivers (per influenza episode), varying as per disease severity, age of patient, and viral type, among other factors. Finally, the health-related quality of life (HRQoL) of caregivers was affected due to emotional and physical overload, with PAR-ENT-QoL scores (from 79.77 at baseline to 60.99 post-Influenza-like Illness), being the severity and duration of the influenza episode factors associated with worse caregiver HRQoL [26].

Seasonal influenza generates a substantial economic impact. Direct costs include outpatient visits, hospitalizations, and management of complications, while indirect costs arise from productivity loss, sick leaves, and caregiver burdens. Total costs can reach up to 56.7 million euros per million inhabitants in industrialized countries [4]. In Spain, influenza has an annual direct cost of approximately €128 million per season, being most of the expenditures (80%) concentrated on individuals over 45 years of age [9].

Other viruses such as respiratory syncytial virus (RSV), which have recently gained greater attention, are a significant cause of respiratory illness across all age groups, with a particularly high impact on young children and older adults. In 2023, the total cost associated with RSV cases (without the vaccine) in Spain was around €1.2 million, mostly driven by patients aged 70–80 years [27]. Although substantial, this figure falls short of the economic burden associated with influenza.

## 3. Role of Influenza Vaccination and Impact of Coverage Reduction

Seasonal vaccination is the most effective strategy to reduce influenza incidence, severity and consequences, particularly in high-risk populations, as well as for preventing influenza-related overload of the healthcare system [12,28]. Beyond direct prevention of influenza, vaccination has a role in preventing dementia, reducing unnecessary use of antibiotics, and in pandemic preparedness. Two systematic reviews found that adult vaccination was associated with a significantly lower risk of dementia, suggesting that the vaccination of older people against influenza may also aid in the prevention of dementia [29,30]. However, further research is still needed to confirm these findings and better understand the underlying mechanisms. Furthermore, increased influenza vaccination coverage is associated with global reductions in antibiotic use. Thus, expanding influenza vaccination could be an important intervention to reduce unnecessary antibiotic prescribing [31,32]. Finally, seasonal influenza vaccination programs can play a crucial role in pandemic preparedness. Evidence from the 2009 A(H1N1) influenza pandemic and the COVID-19 pandemic indicates that countries with established seasonal influenza vaccination infrastructures were better equipped to rapidly deploy vaccines and achieve higher vaccination coverage. Therefore, strengthening seasonal influenza programs during interpandemic periods can help reduce the burden of seasonal influenza while also enhancing healthcare system preparedness, boosting public confidence in vaccinations, and improving responsiveness to future respiratory pandemics [33,34].

In 2003, the WHO advocated for an increase in influenza vaccination coverage to achieve a 75% vaccination coverage rate (VCR) target among risk groups by 2010. Nevertheless, in most European countries, VCRs remained suboptimal in all risk groups during the 2022–2023 influenza season [35]. One study investigated the seasonal influenza vaccination programs across 54 countries and areas in the WHO European Region over a 15-year period (2008/09–2022/23). It detected the expansion of target group recommendations and increased vaccine use in middle-income countries and areas. However, challenges persist, including low vaccination uptake, limited per capita vaccine supplies in lower-resourced settings, and gaps in coverage monitoring [36].

In Spain, influenza vaccination coverage among people >64 years old has remained suboptimal since 2017, with a 58.5% VCR in 2024 [37]. These figures indicate a persistent gap between current coverage and recommended targets, particularly among healthcare professionals and other priority groups. Similarly, Spanish coverage rates in 2024 among healthcare professionals (39.5%) and pregnant woman (60.9%) also remain low [37]. These figures highlight a persistent gap that undermines the potential benefits of vaccination and keeps the WHO target as a distant, unmet goal. The relevance of improving vaccine uptake in Spain becomes clearer when considering its clinical, societal, and economic implications.

### 3.1. Clinical Impact of Influenza Vaccination

Seasonal influenza vaccination substantially reduces the risk of infection and influenza-related complications—including ICU admissions, hospitalization, and mortality, predominantly among high-risk groups, such as young children (<5 years), older adults (>65 years), pregnant women, and immunocompromised individuals [38].

A systematic review and meta-analysis demonstrated the significant effectiveness of vaccination in reducing influenza-related complications across all age groups (<5 years, 5–65 years and >65 years), when compared to the non-vaccinated population [39]. Importantly, vaccination demonstrated a protective effect against mortality, with benefits observed as early as the first month following infection and extending up to almost one year [39]. These benefits were mainly observed among high-risk populations, with vaccination showing effectiveness in reducing mortality risk for all influenza virus infections in patients >65 years old and in patients with comorbidities [39]. During the 2023/24 season (the first nationally recommending vaccination for children 6–59 months), studies showed high adjusted Influenza Vaccine Effectiveness in this age group [40].

According to available data, in Spain, during the seasons 2013–2014 and 2014–2015, influenza vaccination showed a positive impact in reducing disease severity and avoiding complications among the elderly [41]. In particular, influenza vaccination showed significant protection against ICU admission (OR 0.35, 95% CI 0.21–0.59; *p* < 0.001); mechanical ventilation (OR = 0.56, 95% CI 0.39–0.80; *p* = 0.002); secondary bacterial pneumonia (OR = 0.61, 95% CI 0.39–0.98; *p* = 0.04) and a higher degree of dependence (OR = 0.74, 95% CI 0.55–0.99; *p* = 0.04) [41]. Mazagatos et al. quantified an average of 9900 influenza hospitalizations and 1541 ICU admissions averted by vaccination among people ≥65 years old each year, taking into account the 2017–2018 and 2019–2020 seasons. According to these authors, vaccination was able to prevent between 11% and 26% of influenza hospitalizations and around 40% of ICU admissions in the elderly in the three pre-pandemic seasons, even in those with moderate vaccine effectiveness [42]. More recent data showed that influenza vaccination was effective in reducing mortality, influenza incidence, and hospitalizations related to influenza among the elderly in the Catalonia region (Spain) over seven seasons (2014/2015 to 2020/2021) [43].

Furthermore, available evidence shows that vaccination promotes CV risk avoidance, including reductions in CV mortality of up to 41% when given early after acute myocardial infarction. Reflecting this, the European Society of Cardiology (ESC) recognizes vaccination as the fourth pillar of CV prevention, strongly endorsing annual influenza vaccination (class IA) for patients with established CV disease or stable CAD. The 2024 ESC guidelines for chronic coronary syndromes extend recommendations to pneumococcal and COVID-19 vaccination and also propose influenza vaccination during hospitalization for acute coronary syndromes. In patients who have had heart failure, the 2021 ESC guidelines recommend influenza and pneumococcal vaccination to reduce hospitalizations [19].

Finally, maternal influenza vaccination prevented influenza-confirmed hospitalizations in children ≤6 months with a 61% (95% CI: 27–79%) effectiveness [44]. This is particularly important because no influenza vaccines are authorized for children <6 months of age. Taken together, these findings support the role of influenza vaccination in reducing severe outcomes among high-risk populations.

Besides the significant clinical benefits, vaccination alleviates the pressure on healthcare systems by lowering hospitalization rates, saving medical resources, and improving seasonal healthcare service efficiency. It was estimated that achieving a 75% VCR target in Spain could avoid 180,300 influenza cases, 93,500 general practitioner (GP) visits, 4200 hospitalizations, and 1200 deaths, particularly among the elderly [45].

### 3.2. Societal Impact of Influenza Vaccination

Vaccination is also an effective strategy to lower work absenteeism rates and reduce sick leaves. A review by Fisman et al. examined the impact of vaccination on absenteeism and productivity loss among healthy working-age adults. It identified less work time and productivity loss, as well as lower costs, among vaccinated versus unvaccinated individuals, members of households with vaccinated children versus those with unvaccinated children, or during vaccine-mismatched seasons [46]. Given that influenza vaccination reduces both incidence and severity of influenza, a positive impact of vaccination on work and school absenteeism (missed educational opportunities) and productivity loss was not unexpected.

Indeed, children are the main transmitter of the influenza virus [12], playing a key role in the community circulation and amplification of influenza epidemics [40]. The circulation of influenza in the pediatric population entails substantial healthcare, social, and economic costs, primarily due to school absenteeism and parental absenteeism from work [47,48]. In this context, pediatric influenza vaccination is recognized not only for providing individual protection to the child but also for offering protection to the entire community and reducing the incidence of influenza in the general population [12,28,49].

Moreover, vaccinating HCPs reduces absenteeism in a key sector thereby supporting the sustained functioning of a workforce that is critical to public health. By mitigating absenteeism, improving HRQoL, and alleviating caregiver burden, influenza vaccination strengthens not only individual protection but also societal and healthcare system resilience.

Finally, expanding vaccination coverage would have a positive impact on HRQoL in both patients and caregivers. Vaccinated flu adult outpatients reported significantly higher scores on the physical component summary of HRQOL than non-vaccinated counterparts (*p* = 0.0011) [50]. Furthermore, vaccination may reduce family and social stress, particularly in households with vulnerable individuals, due to prolonged illness and longer recovery time, as well as reduce the psychological and physical impact on non-professional caregivers.

### 3.3. Economic Impact of Influenza Vaccination

Several studies have assessed the cost-effectiveness of routine annual influenza vaccination reporting that influenza vaccination is a cost-saving or cost-effective intervention, particularly for high-risk groups, healthcare workers, and in certain contexts, healthy working age adults [51,52,53].

The economic benefits of influenza vaccination derive from both reduced direct costs (hospitalizations, medical consultations, treatment, and use of healthcare resources) and indirect costs (absenteeism, increased productivity, and reduced burden on caregivers).

In Spain, the total economic burden of influenza during the 2021/22 influenza season, was estimated to exceed €109 million (Table 1) [45]. Another study estimated influenza vaccination impact on healthcare costs among the elderly in Catalonia (Spain) in seven seasons (2014/2015 to 2020/2021). It reported savings of €1863.39 per person/flu season vs. non-vaccinated individuals, including all visits and medical procedures related to either influenza or pneumonia [43].

According to modeling data from Fougerolles et al. [45], increasing vaccination coverage from 47% to 75% could have prevented 180,300 additional influenza cases (20% of the total), leading to a total potential saving of €43 million in Spain (€26 million in direct medical costs and €17 million in work absenteeism costs).

Based on model-derived estimates from Fougerolles et al., a 1% reduction in national vaccination coverage in Spain would correspond to more than 6400 additional influenza cases and approximately €1.54 million in added seasonal costs, assuming a linear relationship. As shown in Table 2, older adults (≥65 years) represent by far the most impactful group in economic terms, with each 1% drop in vaccination coverage leading to nearly €1.2 million in additional costs. This reflects the high rates of influenza-related complications requiring medical attention. Individuals with chronic conditions, a group often under-vaccinated despite their increased risk, also represent a significant share of the incremental cost, with an estimated €308,918 per 1% reduction in vaccine coverage. In contrast, while children aged 6–24 months and pregnant women contribute less to the overall financial burden per 1% change in coverage, these populations remain clinically important due to their vulnerability to complications and their role in virus transmission.

These calculations are based on: (1) specific vaccine effectiveness defined for each risk group (Table 1), providing a realistic approximation of the potential benefits of increased vaccination coverage, and (2) current coverage levels, which in most groups are close to or above 50% (Table 1)—thresholds where the protective effect of herd immunity may start to play a role. These estimates should be interpreted within the assumptions of the original model, including the underlying coverage levels, risk-group-specific vaccine effectiveness, and the epidemiological context of the analyzed season. Moreover, the vaccination coverage estimate in children included in Table 1 corresponds to the 2005/2007 season, which may limit the interpretation. Following the implementation of the Spanish recommendations for pediatric influenza vaccination, available data suggest a progressive increase in vaccine uptake among children: from 37.22% coverage in 2023 to 45.56% in 2024, among children 1–5 years old [37].

It is important to note that this estimate may be conservative, as it corresponds to a post-COVID-19 season when influenza virus circulation was still limited.

These calculations illustrate the economic and societal burden associated with low vaccine coverage rates. Each additional percentage point of coverage has a measurable impact—not only in terms of healthcare and productivity costs, but also in the strain on health systems and protecting vulnerable populations. In a context of limited healthcare resources and prioritization of new vaccines (COVID-19, RSV), these estimates reinforce the public health and economic relevance of influenza vaccination in Spain.

## 4. Challenges and Opportunities to Increase Vaccination Coverage in Spain

### 4.1. Current Influenza Vaccination Practices

The vaccination coverage goals for the 2024–2025 season in Spain aimed to achieve a VCR ≥ 75% among the elderly and HCP, and ≥60% VCR in pregnant women and individuals at risk. The Spanish Ministry of Health recommended vaccination against influenza in the 2024–2025 season for the following population groups: (A) Those at higher risk of complications or serious conditions: people >60 years old; people institutionalized >5 years old; people >12 years old with a range of elsewhere defined concomitant diseases; pregnant women and women during the puerperium (up to 6 months after delivery who were not vaccinated during pregnancy); people living with immunosuppressed patients; children between 6 and 59 months of age; children between 5 and 12 years of age with at-risk conditions; children 5–18 years old receiving prolonged treatment with acetylsalicylic acid (due to the possibility of developing Reye’s syndrome after influenza); current smokers; people with celiac disease; and people with cerebrospinal fluid fistula and cochlear implant or awaiting implant. (B) Those belonging to critical and essential services to the community, to reduce the impact and maintenance of the services: staff of public and private health and social-health centers; people working in essential public services, in particular firefighters, state security forces, etc.; trainees in health and social-health centers; nursery and kindergarten staff (<5 years of age); and people with direct occupational exposure to animals or their secretions [28,54].

Despite these comprehensive recommendations and clearly defined vaccination targets, coverage rates for the 2024 season fell short of the established goals. Specifically, in 2024, coverage reached only 58.5% among individuals aged 65 and older, 60.9% among pregnant women, and 39.5% among healthcare personnel [37].

European countries are far from reaching the WHO’s recommended vaccination coverage targets, with the current figures reflecting persistent gaps in vaccine acceptance and delivery. Analysis of VCRs in European countries between the 2013–14 and 2015–16 seasons shows that coverage rates vary dramatically between countries, ranging from 1.1% in Estonia to 74.5% in Scotland among elderly populations, with a median of 35.3% across 21 countries [55].

Although a 75% coverage goal may seem ambitious or even unattainable in the short term, it is important to recognize the significant health and socioeconomic benefits associated with incremental improvements. Each percentage point increase in vaccination coverage translates into a meaningful reduction in influenza cases, healthcare burden, and associated costs, being these incremental improvements well worth pursuing. Sustained efforts to raise awareness, address barriers, and implement targeted interventions are crucial to gradually closing the gap and strengthen population immunity against influenza.

### 4.2. Barriers and Opportunities to Improve Vaccine Uptake

Despite the availability of safe and effective vaccines, as well as established vaccination services and policies, there exists a delay in acceptance or refusal of vaccination, the so-called *vaccine hesitancy*. For instance, routine vaccination is accepted by about half of parents (53.61% of those surveyed agreed with routine vaccination in children under 5 years of age) [12]. Vaccine hesitancy is complex and context-specific, and it varies across time, place, and vaccine type [56,57,58,59]. Barriers to influenza vaccination can be broadly categorized into psychological, physical, contextual, and sociodemographic factors [60].

A lack of adequate knowledge about influenza and its vaccination has been consistently identified as a barrier to vaccine uptake [60]. Inadequate understanding of the virus’s potential complications, the benefits of vaccination, or the populations for whom vaccination is recommended, contribute to low vaccination coverage. In addition to limited awareness, certain psychological factors and misperceptions—such as perceiving influenza as a mild or unimportant illness (“trivialization of the disease”)—may lead individuals to underestimate the seriousness of the infection and its risks, further discouraging vaccination [60]. A qualitative study conducted in Spain found that misinformation and a lack of knowledge led the general population to consider influenza as a common, low-risk illness. Additionally, fear of adverse effects from the vaccine contributed to hesitancy and negative attitudes towards vaccination within the general population [61].

The main determinants of declining seasonal influenza vaccination among pregnant women in Spain included the under-estimation of the risk of contracting or being harmed by influenza, and a lack of information [62].

Burgaya-Subirana et al. conducted research among families with children between 6 months and 14 years of age attending pediatric consultations and found that major reasons cited for not vaccinating include underestimation of the disease (lack of risk perception), fear of unwanted effects (concern about vaccine safety), and lack of information about vaccination [12]. Similarly, a lack of recommendations and considering the influenza vaccine as not necessary were the main reasons for not getting vaccinated among the adult population [63].

Targeted information campaigns aimed at both the general population and particularly at-risk groups along with initiatives that help debunk myths about vaccination may help increase vaccination coverage. The awareness-raising campaigns should be focused on changing the mindset of the benefits/goals of influenza vaccination, from preventing the infection and complications to reducing hospitalizations and deaths, rather than avoiding the flu overall (Table 3).

Awareness of vaccination effectiveness was particularly successful during the COVID-19 pandemic. Higher vaccination rates against influenza were observed after the COVID-19 pandemic [37], supporting the notion that vaccine uptake tends to be higher when there are greater social pressures and perceived benefits, and lower when such pressure is lacking [60].

External contextual factors influencing vaccine uptake include access-related issues (e.g., costs and reimbursement conditions) or organizational factors linked to interactions with the healthcare system. For example, individuals who did not receive direct recommendations from medical personnel were frequently reported to be less likely to vaccinate [60]. In children, the intervention most strongly associated with having children vaccinated in the past was receiving recommendation from a pediatrician (68.18% of vaccinated children). Similarly, a lack of professional recommendation was the main reason for not vaccinating previously [12].

Therefore, it is worth acknowledging the role of HCPs in awareness and increasing vaccination coverage [12]. HCPs beyond primary care should actively recommend the influenza vaccine, ensuring that individuals at risk are appropriately and timely informed. Specialists should not swing the responsibility to patients but take an active role in advising them about vaccination (Table 3). Vaccination of healthcare professionals themselves is also essential, both as an active recommendation and as a strategy for individual protection and for reducing the risk of transmission to patients (Table 3). Finally, the role of occupational medicine in expanding vaccination coverage for individuals aged 60–67 who are active, healthy, and do not visit their Primary Care center on a regular basis should also be strongly considered.

Important factors contributing to suboptimal influenza vaccination coverage among healthcare professionals include a lack of specific training on vaccines in training plans in medical careers and other health careers, a deficit in vaccine culture in the continuous training of adult doctors, and an absence of professional incentives for the promotion of high vaccination coverage [64]. In addition, younger healthcare personnel have a lower perception of risk and lower vaccination rates, and they are less likely to recognize their role as transmission agents [65]. Addressing these barriers through strengthened vaccine education, continuous training initiatives, and targeted institutional strategies may help improve vaccination among healthcare professionals (Table 3).

Emerging respiratory viruses, such as SARS-CoV-2 and RSV, have added complexity to vaccination efforts, as they strain healthcare systems and further emphasize the need for comprehensive vaccination strategies to protect vulnerable groups. Simultaneous vaccination against different agents causing acute respiratory infections could be an attractive strategy to maximize vaccine uptake in vulnerable populations. Co-administration of inactivated influenza vaccines and SARS-CoV-2 vaccines has no detrimental effects on cellular immune responses to SARS-CoV-2 and serological responses to influenza [66]. Likewise, simultaneous vaccination against RSV and influenza show an acceptable safety profile and did not hamper the immunogenicity of either vaccine in individuals ≥65 years [67]. Therefore, either RSV or SARS-CoV-2 vaccines can be administered together with the influenza vaccine to reduce “vaccine fatigue” among vulnerable populations and increase coverage (Table 3).

Accessibility and convenience are also significant factors influencing vaccination coverage. Vaccination rates increase substantially when vaccines are offered through more accessible methods, such as pharmacies, mobile vaccination units, reminder systems (SMS or email), free programs, and extended clinic hours in the evenings or on weekends. These measures are particularly relevant for target groups such as the elderly or people with chronic diseases. These populations frequently experience mobility limitations or difficulties accessing healthcare services. In Spain, expanding community-based vaccination delivery models and improving accessibility could provide additional opportunities to increase vaccination coverage and alleviate the burden of influenza in vulnerable populations (Table 3).

Finally, policymakers play a key role in supporting vaccination efforts in order to approach ideal vaccination coverage goals. Rather than focusing on maintaining the previous year’s vaccination rate, efforts should be directed towards establishing clear and ambitious coverage targets consistent with WHO recommendations. These findings point to the importance of investing in effective influenza vaccination campaigns, strengthening communication around their benefits, and prioritizing resources towards interventions that offer greater public health and economic value (Table 3). However, an important limitation is that the impact of vaccination campaigns in Spain is rarely formally evaluated or published, which restricts opportunities to reassess and refine these strategies in order to improve vaccine coverage and maximize their potential benefits.

A further challenge in Spain stems from the decentralized organization of the healthcare system, which can result in fragmented planning and implementation of vaccination campaigns across autonomous communities. Such heterogeneity may contribute to differences in access, communication approaches, and vaccine uptake, thereby complicating efforts to achieve consistent and equitable population protection nationwide.

## 5. Conclusions

Seasonal influenza continues to represent a relevant public health challenge in Spain, generating a substantial clinical, societal, and economic burden, particularly among older adults and other high-risk populations. Despite the availability of effective vaccines and longstanding recommendations for immunization, vaccination coverage remains below target levels across several priority groups.

Available evidence consistently supports the role of influenza vaccination in reducing severe outcomes, healthcare utilization, and associated economic and societal impact. In addition, previously published modeling analyses suggest that even modest improvements in vaccination coverage may translate into meaningful health and economic benefits at the population level. In this regard, model-based estimates indicate that increasing vaccination coverage to 75% in Spain could have prevented approximately 180,300 influenza cases, equivalent to 20% of the total, and generated potential savings of €43 million, including €26 million in direct medical costs and €17 million in work absenteeism costs.

Addressing current coverage gaps will likely require a multifaceted approach, including clearer and more ambitious coverage targets, improved communication strategies, stronger engagement of healthcare professionals, and stronger performance of vaccination campaigns. Greater coordination across autonomous communities may also help reduce variability in implementation and improve equitable access to vaccination services. Establishing a unified national influenza vaccination data monitoring platform and developing standardized promotional materials and vaccination service protocols applicable to all regions may improve coordination between autonomous communities.

Overall, strengthening influenza vaccine uptake should be considered an important public health priority in Spain. Enhancing vaccination strategies may contribute to reducing the burden of influenza and improving the resilience of the healthcare system.

## Figures and Tables

**Table 1:** Estimated influenza-related costs by risk group in Spain. Adapted from Fougerolles et al. [45].

Risk Group	At-Risk Population, *n* (% of Eligible Population)	VCR (Season) * Weighted VCR	Prevented Cases at This VCR, *n* (% of Total Prevented Cases)	Influenza-Related Costs Averted at Real VCR
At-risk population (total)	18,585,180 (100)	47% * (2021/2022)	344,900 (100)	€109,308,000
Older adults (aged ≥ 65 years)	9,526,631 (51.26)	69% (2021/2022)	285,600 (82.81)	€83,514,527
Children aged 6–24 months	513,993 (2.77)	7% (2005/2007)	3000 (0.87)	€138,374
Individuals with chronic conditions *	7,342,110 (39.51)	17% (2011/2012)	37,200 (10.79)	€21,924,839
Pregnant women	297,828 (1.60)	55% (2021/2022)	4400 (1.28)	€551,892
Healthcare workers	904,619 (4.87)	61% (2021/2022)	14,700 (4.26)	€3,178,423

VCR: vaccination coverage rate. * Chronic conditions included: diabetes mellitus and other metabolic diseases, chronic cardiopathy, cancer, chronic lung diseases, chronic inflammatory diseases and bowel malabsorption syndromes, pathologies associated with an increased risk of aspiration of respiratory secretions, chronic respiratory disease, chronic liver diseases, chronic neurological disease (including stroke/transient ischemic attack, cerebral palsy/multiple sclerosis), chronic renal failure/adrenal insufficiency, human immunodeficiency virus and immunodepression, hemopathies and hemoglobinopathies, asplenia or dysfunction of the spleen, morbid obesity, and pathologies for which major surgical interventions are planned.

**Table 2:** Model-based incremental costs per 1% decrease in vaccination coverage by risk group in Spain.

Risk Group	Incremental Number of Influenza Cases per 1% Reduction in VCR	Incremental Costs per 1% Reduction in VCR
At-risk population (total)	6439	€1,540,136
Older adults (aged ≥ 65 years)	4920	€1,176,709
Children aged 6–24 months	8	€1950
Individuals with chronic conditions *	1292	€308,918
Pregnant women	33	€7776
Healthcare workers	187	€44,784

VCR: vaccination coverage rate. * Chronic conditions included: diabetes mellitus and other metabolic diseases, chronic cardiopathy, cancer, chronic lung diseases, chronic inflammatory diseases and bowel malabsorption syndromes, pathologies associated with an increased risk of aspiration of respiratory secretions, chronic respiratory disease, chronic liver diseases, chronic neurological disease (including stroke/transient ischemic attack, cerebral palsy/multiple sclerosis), chronic renal failure/adrenal insufficiency, human immunodeficiency virus and immunodepression, hemopathies and haemoglobinopathies, asplenia or dysfunction of the spleen, morbid obesity, and pathologies for which major surgical interventions are planned. Minor discrepancies in totals are due to rounding.

**Table 3:** Proposed measures to improve accessibility and uptake of seasonal influenza vaccines in Spain.

**1. Expand vaccination points**
Consider non-medical sites for influenza vaccination, such as retail (pharmacy) sites
Consider extended vaccination hours in the evenings or weekends
Implement workplace vaccination or school-based vaccination programs
Implement vaccination programs in nursing homes or institutions for people with higher risk
Establish mass vaccination sites during vaccination campaign
**2. Expand vaccination opportunities**
Implement opportunistic vaccination
Consider administering influenza vaccine together with RSV or SARS-CoV-2 vaccines to reduce “vaccine fatigue”
Provide accessible vaccination points for people with mobility limitations or difficulties accessing healthcare services
**3. Reinforce active recruitment**
Use digital reminder–recall systems to send pre-booked appointments for eligible patients
**4. Reinforce the commitment of HCPs and professional societies**
Strengthen HCP roles in increase coverage, since professional recommendation is identified as the main reason for vaccinating
Enhance healthcare organizations’ ethical commitment to at-risk patients.
**5. Increase information and communication**
Create tailored educational materials: materials to schools, nurseries, and workplaces, adapted to different languages and cultural contexts
Implement targeted campaigns aimed at both the general population and at-risk groups: wide and transparent communication strategies targeting long-term benefits of vaccination, benefits of vaccination beyond flu prevention, and debunk myths about vaccination
Implement information points/lines where answer questions, doubts and concerns about flu and vaccination
Increase risk perception among the general population (not at risk)
**6. Increase commitment of policy makers**
Establish clear and ambitious coverage targets consistent with WHO recommendations
Prioritize resources towards public health interventions
Increase investments and resources in effective influenza vaccination, education, and communication campaigns
Establish a unified Spanish influenza vaccination data monitoring platform
Improve coordination between autonomous communities in planning and implementation of vaccination campaigns and promotional materials

HCP: healthcare professionals; WHO: world health organization.

## Data Availability

No new data were generated for this study. All information presented is based on previously published studies and publicly available sources, which are cited in the manuscript.

## Abbreviations

The following abbreviations are used in this manuscript:

CAD	Coronary artery disease
COPD	Chronic obstructive pulmonary disease
CV	Cardiovascular
ECDC	European Centre for Disease Prevention and Control
ESC	European Society of Cardiology
HCP	Healthcare professional
HRQoL	Health-related quality of life
ICU	Intensive care unit
ILI	Influenza-like illness
QALY	Quality-adjusted life year
RSV	Respiratory syncytial virus
